# The Protective Effects of Glycyrrhetinic Acid and Chrysin against Ischemia-Reperfusion Injury in Rat Ovaries

**DOI:** 10.1155/2018/5421308

**Published:** 2018-05-14

**Authors:** Rauf Melekoglu, Osman Ciftci, Sevil Eraslan, Saadet Alan, Nese Basak

**Affiliations:** ^1^Faculty of Medicine, Department of Obstetrics and Gynecology, University of Inonu, 44280 Malatya, Turkey; ^2^Faculty of Medicine, Department of Medical Pharmacology, University of Inonu, 44280 Malatya, Turkey; ^3^Department of Obstetrics and Gynecology, Elbistan State Hospital, 46300 Kahramanmaras, Turkey; ^4^Faculty of Medicine, Department of Pathology, University of Inonu, 44280 Malatya, Turkey; ^5^Faculty of Pharmacy, Department of Pharmaceutical Toxicology, University of Inonu, 44280 Malatya, Turkey

## Abstract

**Objective:**

To evaluate the protective effects of glycyrrhetinic acid (GA) and chrysin (CH) on experimental ischemia-reperfusion (I/R) injury in rat ovaries using tissue oxidative stress marker levels, hormone levels, and histopathological scores.

**Methods:**

Sixty healthy rats were randomly divided into six equal groups: control, I/R, I/R + CH (50 mg/kg/day), I/R + GA (100 mg/kg/day), CH (50 mg/kg/day), and GA (100 mg/kg/day). Biochemical, hormonal, and histopathological evaluations were performed on blood and tissue samples 14 days after CH and GA treatment.

**Results:**

The antioxidant defense system parameters were significantly higher in the ovarian tissues of the I/R + CH and I/R + GA groups than in those of the I/R group. Serum follicle-stimulating hormone levels were significantly reduced, and serum anti-Müllerian hormone levels were significantly increased in rats treated with CH and GA compared with those in the I/R group. Additionally, the histopathological scores of the I/R + CH and I/R + GA groups were significantly improved compared with those of the I/R group.

**Conclusions:**

The significant improvements in tissue oxidative stress parameters, serum hormone levels, and histological scores observed in this study indicate that treatment with CH or GA may be a conservative approach to prevent I/R injury in adnexal torsion cases after the ovarian detorsion procedure.

## 1. Introduction

Ovarian torsion, defined as twisting of the ovary and its vascular peduncle around the axis of the suspensory ligament, accounts for nearly 3% of all gynecological emergencies [1]. Conditions that lead to ovarian enlargement, such as adnexal cysts, ovarian hyperstimulation, or pregnancy, and hyperlaxity of the ovary proprium and infundibulopelvic ligaments are considered risk factors for ovarian torsion [2]. Although it can occur in women of all ages, it is primarily seen in women of reproductive age [3]. Depending on the degree of torsion, venous return to the ovarian tissue decreases, and stromal edema and internal hemorrhage subsequently occur. If arterial blood flow ceases, ischemic and necrotic processes begin in the tissue. Early diagnosis and management are essential to protect ovarian function [4]. Recent studies have recommended conservative treatment with detorsion to preserve the twisted ovary even when it seems cyanotic. This detorsion procedure does not significantly increase the risk of pulmonary embolism, and oophorectomy is no longer preferred [5]. However, detorsion of the twisted ovary leads to another risk, ischemia/reperfusion (I/R) injury, which is related to reperfusion and neutrophil infiltration of the tissue. As a result of the reperfusion process, an excessive amount of molecular oxygen supplementation to the ovarian tissue occurs, and the production of reactive oxygen species (ROS) increases. These ROS cause cellular damage by attacking cellular membranes through the peroxidation of polyunsaturated fatty acids [6]. Several antioxidants have been found to be effective in the prevention of oxidative injury and inflammation in ovarian tissues exposed to I/R injury [7].

Chrysin (CH) is a natural flavonoid found in honey, propolis, and many plant extracts. Numerous studies have demonstrated the antioxidant, anti-inflammatory, and antidiabetogenic effects of chrysin [8]. Glycyrrhetinic acid (GA) is an aglycone saponin extracted from licorice root that has been widely used for its well-known anti-inflammatory, antitumor, and antihepatitic effects [9]. The benefits of these antioxidants have been demonstrated in I/R injuries in the heart and brain, but no studies have been conducted on the effects of CH or GA on I/R injury in ovarian tissue.

The objective of this study was to evaluate the protective effects of GA and CH in experimental I/R injury in rat ovaries using histopathological scores, biochemical lipid peroxidation markers, and hormonal assessment.

## 2. Materials and Methods

### 2.1. Chemicals

Chrysin was obtained from MP Biomedicals, LLC (Santa Ana, CA, USA). Glycyrrhetinic acid was obtained from Sigma Chemical Co. (St. Louis, MO, USA). All other chemicals for biochemical and histological analysis were purchased from Sigma Chemical Co. and were of analytical grade or the highest grade available.

### 2.2. Animals and Treatment

Experiments were performed under the animal ethics guidelines of the Inonu University Institutional Animals Ethics Committee (Approval No. 2016-A32). Sixty healthy adult female Wistar albino rats aged 3-4 months, obtained from the Experimental Animal Institute (Malatya, Turkey), were used in this experiment. Animals were housed in sterilized polypropylene rat cages under a 12/12-h light/dark cycle at an ambient temperature of 21°C. Food and water were provided ad libitum. Rats were randomly divided into six equal groups as follows (*n* = 10 per group): control (sham-operated), I/R, I/R + CH (50 mg/kg/day), I/R + GA (100 mg/kg/day), CH (50 mg/kg/day), and GA (100 mg/kg/day). CH and GA were dissolved in corn oil and orally administered for 14 days by gavage. The dose and treatment period of GA and CH were chosen based on previous studies [10, 11]. After the treatment course, the animals were euthanized without pain or distress using increasing doses of anesthetic (intraperitoneal lethal doses of pentobarbital; Bioveta, Inc., Ankara, Turkey), and the ovaries were removed for histopathological analyses. Blood samples were collected from the left ventricle with an injector under anesthesia. Tissues samples were stored at −45°C in a deep freezer until the analyses were performed.

### 2.3. Surgical Procedures

All surgical interventions were carried out in a proper laboratory environment under sterile conditions. After the rats were weighed, ketamine (50 mg/kg) and xylazine (10 mg/kg) were injected intramuscularly for anesthesia. The rats were then placed in a dorsally recumbent position, and the surgical area was disinfected and dressed with sterile coverings. Surgery was performed at 36 ± 1°C ambient temperature to prevent hypothermia. The abdomen was entered by making a longitudinal midline incision of approximately 2 cm in the subabdominal area to reveal the ovaries. The I/R model was constructed by placing vascular clamps approximately 1 cm under the adnexal structure to simulate ovarian torsion. After 2 h of simulated torsion, the vascular clamps were opened to allow reperfusion, and the incision line was closed with 4-0 nylon sutures.

### 2.4. Biochemical Assay

Tissues were homogenized using a Teflon glass homogenizer in 150 mM KCl (pH 7.4) at a 1 : 10 (w/v) dilution of the whole homogenate. The homogenates were centrifuged at 18,000 ×g at 4°C for 30 min to determine the malondialdehyde (MDA) and reduced glutathione (GSH) concentrations and superoxide dismutase (SOD) and catalase (CAT) activities or at 25,000 ×g for 50 min to determine glutathione peroxidase (GPx) activity. The levels of MDA, an index of lipid peroxidation, were determined via thiobarbituric acid reaction using the method described by Yagi [12]. The product was evaluated spectrophotometrically at 532 nm, and the results are expressed as nmol/g tissue. The method of Sedlak and Lindsay was used to determine the reduced GSH content of the ovarian homogenate at 412 nm [13]. The results are expressed as nmol/mL tissue. GPx activity was determined according to the method developed by Paglia and Valentine [14]. In the presence of GSH reductase and nicotinamide adenine dinucleotide phosphate (NADPH), oxidized glutathione is immediately converted to its reduced form in conjunction with the oxidation of NADPH to NADP. The decrease in absorbance at 340 nm was measured, and GPx activity is presented as U/mg protein. Copper-zinc SOD activity was measured as the inhibition of nitroblue tetrazolium reduction as a result of oxygen generation by the xanthine/xanthine oxidase system [15]. The resulting products, which were evaluated at 560 nm, are expressed as U/mg protein. The CAT activity of the tissues was measured according to the method of Aebi [16]. The enzymatic decomposition of hydrogen peroxide was monitored as the decrease in absorbance at 240 nm. Enzyme activities are expressed as kU/mg protein. The method established by Lowry et al. using bovine serum albumin as a standard was used to determine tissue protein content [17].

### 2.5. Hormonal Assay

Estradiol (E_2_), follicle-stimulating hormone (FSH), luteinizing hormone (LH), and anti-Müllerian hormone (AMH) were quantitatively estimated in rat serum samples using enzyme-linked immunosorbent assay (ELISA) kits (catalog numbers: SL0268Ra, SL0297Ra, SL1093Ra, and SL0504Ra, resp.; Sunlong Biotech Co., Ltd., Zhejiang, China). The E_2_ assay measured concentrations in the range of 5–150 pg/mL, with manufacturer-specified interassay coefficients of variation (CV) of 7.5% and intra-assay CVs of 5.4%. The FSH assay measured concentrations from 0.5 to 10 IU/L; the serum inter- and intra-assay CVs were 6.4 and 4.3%, respectively. The LH assay measured concentrations to 7 IU/L with a minimum detectable concentration of 0.1 IU/L. The serum inter- and intra-assay CVs were 6.8 and 2.8%, respectively. The AMH assay measured concentrations with an assay range of 0.5–20 ng/mL; the manufacturer-specified mean interassay CV was 6.5%, and the mean intra-assay CV was 4.0%.

### 2.6. Histopathological Examination

The ovarian tissues were maintained in 10% formaldehyde solution for histopathological examination. Following routine procedures, embedded tissues were cut into 5 *μ*m thick sections and stained with hematoxylin and eosin (H&E). Tissue damage was histopathologically assessed for hemorrhage, vascular congestion, edema, polymorphonuclear leukocyte (PMNL) infiltration, and apoptosis. At least five microscopic regions were examined to score the specimens semiquantitatively. Each sample was scored for each criterion using a scale ranging from 0 to 3 (0, none; 1, mild; 2, moderate; 3, severe). Total scores were calculated from these parameters. Histopathological evaluations were performed using a light microscope (Olympus BX51; Olympus Corporation, Tokyo, Japan).

### 2.7. Statistical Analysis

All values are presented as the mean ± standard deviation. Differences were considered significant at *P* < 0.01. SPSS software (ver. 18.0; SPSS, Inc., Chicago, IL, USA) was used to perform statistical analyses. The biochemical values were analyzed using one-way ANOVA and Tukey's post hoc honestly significant difference test. Histological results were compared using the Kruskal–Wallis variance analysis. When differences among the groups were detected, group means were compared using the Mann–Whitney *U* test.

## 3. Results

### 3.1. Biochemical Results

The antioxidant (SOD, CAT, GPx, and GSH) and oxidant (MDA) parameters in the rat ovaries among different treatment groups are presented in Table 1. The I/R group had significantly higher ovarian tissue MDA levels than the control group had (*P* < 0.001), whereas the GSH levels and SOD, GPx, and CAT activities were significantly lower in the I/R group than in the control group. MDA levels were significantly decreased, whereas GSH levels and CAT and SOD activities were significantly increased in ovarian tissues in the I/R + CH and I/R + GA groups compared with the I/R group. Treatment with CH or GA in the absence of I/R injury improved tissue oxidative stress markers, such as GSH levels and SOD and GPx activities.

### 3.2. Histological Results

**Figure 1:**
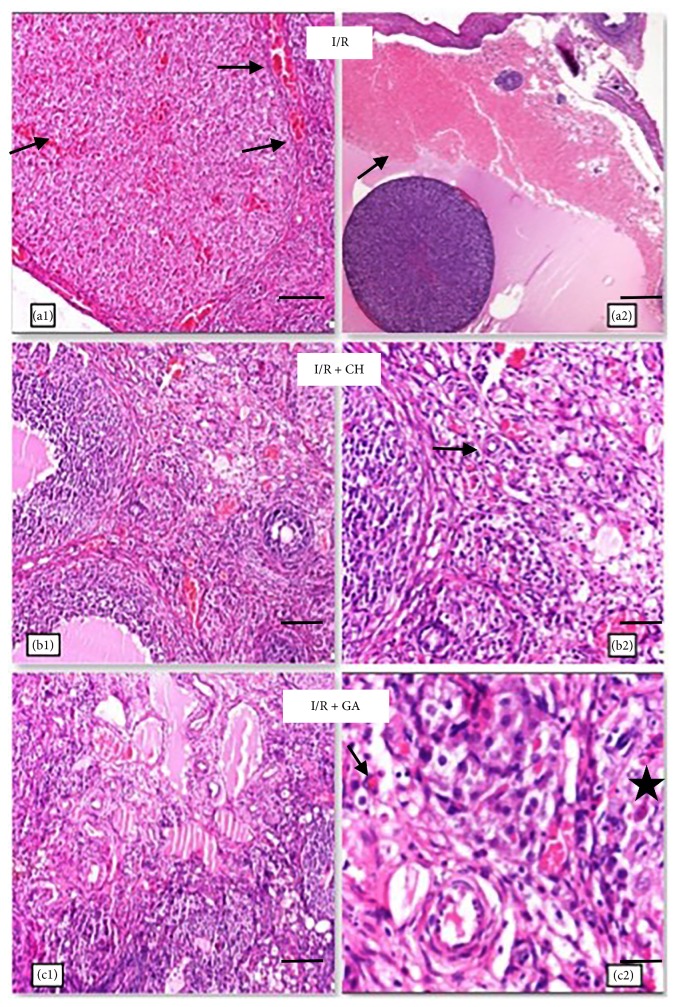
(a1, a2): ischemia-reperfusion (I/R) group. (a1) Vascular congestion indicated by ↑ symbol. (a2) Hemorrhage indicated by ↑ symbol ((a1): hematoxylin and eosin (H&E), 100x, scale bar = 100 *μ*m; (a2): H&E, 40x, scale bar = 400 *μ*m) (b1), (b2): I/R + chrysin (CH) group. Decreased vascular proliferation indicated by ↑ symbol ((b1): HE, 100x, scale bar = 200 *μ*m; (b2): HE, 200x, scale bar = 100 *μ*m). (c1), (c2): I/R + glycyrrhetinic acid (GA) group. Reduced PMNL infiltration is indicated by ↑ symbol, and I/R-induced apoptosis is indicated by *▯* symbol. ((c1): HE, 100x, scale bar = 100 *μ*m; (c2): HE, 400x, scale bar = 25 *μ*m).

The histopathological scores for all six groups are listed in Table 2. Vascular congestion, hemorrhage, edema, PMNL infiltration, and apoptosis were significantly higher in the I/R group than in the control group. All histological parameters were significantly improved with the administration of CH or GA. The anti-inflammatory effects of GA and CH were also shown in the treatment group without I/R injury. The ovarian histopathological changes for I/R, I/R + CH, and I/R + GH are shown in Figure 1.

### 3.3. Hormonal Assessment

The serum E_2_ and AMH levels were significantly lower, and the serum FSH and LH levels significantly higher, in the I/R group than in the control group. The results indicated a significant reduction in serum FSH and LH levels in rats treated with CH or GA compared with the I/R group. Additionally, the serum levels of E_2_ and AMH in rats treated with CH and GA were significantly higher than those of the I/R group. The effects of CH and GA on ovarian reserve markers are shown in Table 3.

## 4. Discussion

In this study, we examined the ovarian protective effects of GA and CH in a rat model of ovarian I/R injury. We demonstrated that I/R injury alone caused a marked decrease in ovarian reserve, which was associated with increased tissue oxidative stress, impaired hormonal changes, and increased histopathological damage. In contrast, the tissue oxidative stress parameters, ovarian reserve markers, and histopathological changes were significantly ameliorated in rats that received GA or CH after I/R injury. Ovarian detorsion without oophorectomy can protect ovarian function, but prophylactic measures are required against I/R injury after the procedure. The exact mechanism of cellular damage after I/R remains unclear. The induction of apoptosis by damaged mitochondrial proteins as a result of ischemia, activation of chemotaxis, and endothelial adhesion of leukocytes by impaired membrane proteins and phospholipids owing to the lipid peroxidation effects of ROS have been proposed as mechanisms in the pathophysiology [18]. ROS induces cellular damage through the overproduction of oxygen free radicals such as superoxide anions (O_2_^−^), hydrogen peroxide (H_2_O_2_^−^), hypochlorous acid (HOCl^−^), and hydroxyl radicals (OH^−^) [19]. For this reason, several studies have focused on pharmaceutical agents with antioxidant and/or anti-inflammatory effects to prevent ovarian I/R injury in animal models [20, 21]. To the best of our knowledge, this is the first study to use CH and GA to treat I/R injury in ovarian torsion, and our results suggest that both of these pharmaceutical agents have protective effects against I/R injury.

The exact mechanism of the antioxidative and anti-inflammatory effects of CH and GA is unclear. The antioxidative properties of CH have been attributed to the inhibition of inducible nitric oxide synthase and cyclooxygenase-2 expression, and the inhibition of proinflammatory nuclear factor kappa B (NF-*κ*B) activity has been suggested as the mechanism for the anti-inflammatory effects of CH [22]. It has also been proposed that the antioxidative and anti-inflammatory effects of GA may be attributed to inhibition of the complement pathway and the suppression of lipopolysaccharide-induced tumor necrosis factor alpha production and NF-*κ*B activation [23]. The results of this study also confirmed the antioxidative and anti-inflammatory effects of CH and GA by demonstrating their protective effects against I/R injury in rat ovaries, such as reducing lipid peroxidation, increasing antioxidant activity, and improving histopathological scores.

MDA is one of the end products of lipid peroxidation, and increased MDA levels reflect oxidative stress. In contrast, elevated GSH levels and increased SOD, CAT, and GPx activities indicate tissue healing after oxidative damage [24]. In this study, we demonstrated a significant improvement in tissue oxidative stress markers MDA, GSH, SOD, CAT, and GPx. Consistent with our results, Yao et al. reported that 75 mg/kg/day CH was effective in preventing focal cerebral I/R injury, and they found that SOD activity significantly increased, and MDA content significantly decreased, in the CH treatment group after I/R injury [25]. Yildirim et al. investigated the effects of octreotide treatment for the prevention of I/R injury in rat ovaries, and they attributed decreased tissue MDA levels to the antioxidant properties of octreotide [26].

Our results also revealed that CH and GA had beneficial effects on ovarian functional restoration after I/R injury. AMH and E_2_ levels were significantly increased, and FSH and LH levels significantly decreased, in the CH and GA treatment groups compared with the I/R injury group. Experimental studies on the effects of I/R injury and antioxidants on ovarian reserve markers are limited. Özcan et al. evaluated the effects of resveratrol against oxidative damage stimulated by cisplatin administration on ovarian reserve in rats. They found that resveratrol treatment significantly increased AMH levels compared with those in the control group [27]. Sahin Ersoy et al. compared the effects of N-acetylcysteine (NAC) and enoxaparin on ovarian tissue preservation by assessing ovarian reserve and oxidative stress markers following ovarian torsion/detorsion injury. They noted that NAC administration resulted in more preantral follicles and significantly higher AMH levels compared with enoxaparin treatment and I/R injury [28].

In the present study, we showed that CH and GA treatment ameliorated histological changes such as vascular congestion, hemorrhage, edema, and inflammatory cell infiltration in ovarian tissue following 2 h of clamping ovarian vessels with atraumatic vessel clips to stimulate ovarian torsion. Tang et al. showed the beneficial effects of glycyrrhizin (which includes GA) on endotoxin-induced acute liver injury after partial hepatectomy in rats. They reported significantly improved histological scores in the liver following glycyrrhizin treatment [29]. Similarly, beneficial effects of sildenafil, montelukast, clotrimazole, and infliximab have been shown in many studies of I/R injury in rat ovaries due to the antioxidant and anti-inflammatory properties of these agents. All of these pharmacological agents have been associated with improvements in histopathological parameters such as vascular congestion, edema, and leukocyte infiltration [30–33].

The main limitation of this study was that only a single dose of CH and GA was examined; different doses of these agents may have different effects. Additionally, we did not perform a preliminary study, and the duration and administration routes of CH and GA were determined based on previous studies. This failure to examine the optimal doses and duration for CH and GA treatments may be addressed in future studies.

## 5. Conclusion

In conclusion, treatment of ovarian I/R injury with CH and GA was effective in reducing ovarian damage. The significant improvements in tissue oxidative stress markers, serum hormone levels, and histopathological scores detected in this study indicate that treatment with CH or GA may be a conservative approach for adnexal torsion cases after the detorsion procedure to prevent I/R injury. The beneficial effects of these agents may be attributed to the antioxidant and anti-inflammatory effects of CH and GA.

## Figures and Tables

**Table 1:** Levels of oxidant and antioxidant parameters in rat ovarian tissues among the treatment groups.

Group	MDA (nmol/g tissue)	GSH (nmol/mL tissue)	SOD (U/mg protein)	GPx (U/mg protein)	CAT (kU/mg protein)
Control	6.38 ± 1.12^a^	55.4 ± 2.66^f^	80.1 ± 10.2^k^	596.4 ± 45.9^p^	0.014 ± 0.001^v^
I/R	9.16 ± 0.98	35.5 ± 2.87	57.6 ± 14.7	468.9 ± 92.6	0.005 ± 0.001
I/R + CH	5.02 ± 1.05^b^	45.2 ± 3.56^g^	75.1 ± 15.1^l^	595.0 ± 67.9^r^	0.010 ± 0.004^x^
I/R + GA	5.14 ± 1.23^c^	50.9 ± 3.44^h^	78.3 ± 12.6^m^	608.8 ± 52.3^s^	0.011 ± 0.005^y^
CH	5.94 ± 1.09^d^	59.5 ± 3.82^i^	83.1 ± 11.5^n^	612.4 ± 79.1^t^	0.011 ± 0.001^w^
GA	6.23 ± 1.34^e^	54.7 ± 4.12^j^	89.9 ± 18.3^o^	626.3 ± 88.4^u^	0.012 ± 0.002^z^

^a^ *P* < 0.001, ^*b*^*P* < 0.001, ^c^*P* = 0.002, ^f^*P* = 0.005, ^g^*P* = 0.003, ^h^*P* < 0.001, ^k^*P* < 0.001, ^l^*P* < 0.001, ^m^*P* < 0.001, ^p^*P* = 0.008, ^r^*P* = 0.008, ^s^*P* = 0.007, ^v^*P* = 0.003, ^x^*P* < 0.001, ^y^*P* < 0.001 compared with I/R group; ^d^*P* = 0.019, ^e^*P* = 0.071, ^i^*P* = 0.007, ^j^*P* = 0.089, ^n^*P* = 0.045, ^o^*P* = 0.008, ^t^*P* = 0.037, ^u^*P* = 0.006, ^w^*P* = 0.065, ^z^*P* = 0.068 compared with control group; MDA, malonaldehyde; GSH, glutathione; SOD, superoxide dismutase; GPx, glutathione peroxidase; CAT, catalase; I/R, ischemia–reperfusion; CH, chrysin; GA, glycyrrhetinic acid.

**Table 2:** Histopathological scores among treatment groups.

Histopathological score (mean ± SD)	Hemorrhage	Edema	Vascular congestion	PMNL	Vascular proliferation	Apoptosis
Control	0 ± 0^a^	0.5 ± 0.1^f^	0.75 ± 0.1^k^	0.75 ± 0^p^	1 ± 0.5^u^	0.25 ± 0.1^*α*^
I/R	2.5 ± 0.5	3 ± 0	3 ± 0	3 ± 0	3 ± 0	1 ± 0.5
I/R + CH	0 ± 0^b^	1 ± 0^g^	1.25 ± 0.5^l^	0.25 ± 0.1^q^	0.5 ± 0^v^	0.5 ± 0.5^*β*^
I/R + GA	0 ± 0^c^	1 ± 0^h^	1.25 ± 0.5^m^	0 ± 0^r^	1 ± 0.2^x^	0.75 ± 0.5^*γ*^
CH	0 ± 0.1^d^	0.5 ± 0.2^i^	1 ± 0^n^	1 ± 0^s^	1.25 ± 0.25^y^	0.75 ± 0.2^*λ*^
GA	0 ± 0^e^	0.75 ± 0.1^j^	1.25 ± 0^o^	1.75 ± 0.5^t^	2 ± 0^z^	0.25 ± 0.2^*ψ*^

^a^ *P* = 0.001, ^b^*P* < 0.001, ^c^*P* < 0.001, ^f^*P* < 0.001, ^g^*P* < 0.001, ^h^*P* < 0.001, ^k^*P* < 0.001, ^l^*P* = 0.001, ^m^*P* = 0.001, ^p^*P* < 0.001, ^q^*P* < 0.001, ^r^*P* < 0.001, ^u^*P* < 0.001, ^v^*P* < 0.001, ^x^*P* < 0.001, ^*α*^*P* = 0.003, ^*β*^*P* = 0.008, ^*γ*^*P* = 0.007 compared with I/R group, ^d^*P* = 0.748, ^e^*P* = 1.000, ^i^*P* = 0.950, ^j^*P* = 0.089, ^n^*P* = 0.645, ^o^*P* = 0.037, ^s^*P* = 0.527, ^t^*P* = 0.009, ^y^*P* = 0.665, ^z^*P* = 0.009, ^*λ*^*P* = 0.003, ^*ψ*^*P* = 0.954 compared with the control group; I/R, ischemia-reperfusion; CH, chrysin; GA, glycyrrhetinic acid; PMNL, polymorphonuclear leukocytes.

**Table 3:** Serum hormone levels among the treatment groups.

Group	E_2_ (pg/mL)	FSH (IU/L)	LH (IU/L)	AMH (ng/mL)
Control	73.5 ± 5.56^a^	2.22 ± 0.31^f^	3.24 ± 0.13^k^	7.54 ± 0.77^p^
I/R	33.2 ± 3.61	5.25 ± 1.00	4.76 ± 0.18	3.47 ± 0.54
I/R + CH	66.7 ± 5.25^b^	3.94 ± 0.48^g^	3.13 ± 0.36^l^	5.36 ± 0.31^r^
I/R + GA	62.9 ± 3.79^c^	3.98 ± 0.28^h^	3.10 ± 0.11^m^	5.85 ± 0.60^s^
CH	74.5 ± 6.69^d^	3.29 ± 0.44^i^	3.11 ± 0.11^n^	7.81 ± 0.74^t^
GA	71.3 ± 6.18^e^	2.71 ± 0.28^j^	3.24 ± 0.04^o^	7.81 ± 0.32^u^

^a^ *P* < 0.001, ^b^*P* < 0.001, ^c^*P* < 0.001, ^f^*P* < 0.001, ^g^*P* = 0.002, ^h^*P* = 0.001, ^k^*P* = 0.004, ^l^*P* = 0.003, ^m^*P* = 0.003, ^p^*P* < 0.001, ^r^*P* = 0.007, ^s^*P* = 0.005 compared with the I/R group, ^d^*P* = 0.848, ^e^*P* = 0.570, ^i^*P* = 0.042, ^j^*P* = 0.095, ^n^*P* = 0.754, ^o^*P* = 0.954, ^t^*P* = 0.043, ^u^*P* = 0.043 compared with the control group; I/R: ischemia-reperfusion; CH: chrysin; GA: glycyrrhetinic acid; E_2_: estradiol, FSH: follicle-stimulating hormone; LH: luteinizing hormone; AMH: anti-Müllerian hormone.
